# Humidity Sensor Based on rGO-SDS Composite Film

**DOI:** 10.3390/mi13040504

**Published:** 2022-03-24

**Authors:** Cheng Lei, Junna Zhang, Ting Liang, Ruifang Liu, Zhujie Zhao, Jijun Xiong, Kai Yin

**Affiliations:** 1 State Key Laboratory of Dynamic Measurement Technology, North University of China, Taiyuan 030051, China; leicheng@nuc.edu.cn (C.L.); nuczjn@163.com (J.Z.); s2006222@st.nuc.edu.cn (Z.Z.); xiongjijun@nuc.edu.cn (J.X.); 2 State Key Laboratory of Bioelectronics, Southeast University, Nanjing 210096, China; lrfnuc@163.com; 3 Department of Energy Technology, Aalborg University, 9220 Aalborg, Denmark; kyi@et.aau.dk

**Keywords:** rGO-SDS, humidity, test, sensitivity

## Abstract

Based on the humidity testing requirements in different environments, this paper investigates the humidity sensitivity of reduced graphene oxide (rGO)-sodium dodecyl sulfate (SDS) composite film humidity sensor. In the experiments, rGO-SDS dispersions with a concentration of 5 mg/mL were prepared, and a microelectromechanical system (MEMS) process was used to prepare the interdigital electrodes. The dispersions were then drop-coated on the interdigital electrodes and dried on a heated plate at 100 °C. The surface characteristics of the rGO-SDS films transferred onto SiO_2_-Si substrates were analyzed by scanning electron microscopy, raman spectroscopy and atomic force microscopy, infrared spectroscopy, X-ray photoelectron spectroscopy, and tested by a correlation system, which showed a linear relationship between humidity variation and the resistance variation of the sensor in the ambient humidity range of 25–95% RH. At room temperature, the linearity of the sensor is about 0.98431 and the sensitivity is about 11.41432 Ω/% RH. At 100 °C, the correlation of the sensor is about 0.95046 and the sensitivity is about 1.0145 Ω/% RH; with a response time of only 9 s at ambient humidity from 25% RH to 95% RH, the sensor has very good repeatability and stability.

## 1. Introduction

A humidity sensor is a device used to measure changes in humidity in an atmospheric environment. It plays an important role in industrial [1], environmental [2,3] and manufacturing [1,4], and health care [5] fields. In recent years, with the continuous development of microelectromechanical system (MEMS) technology, humidity sensors tend to be intelligent, miniaturized and integrated. At present, humidity sensors can be divided into surface acoustic wave type [6,7], quartz crystal microbalance type [8], optical fiber type [9], capacitive type [10,11,12], and resistive type [13,14,15]. Among them, resistive sensors are widely used because of their ease of fabrication, simplicity, low cost, and high measurement accuracy. Humidity sensors work by directly or indirectly adsorbing water molecules from the measurement environment through the adsorption effect, which causes changes in the chemical or physical properties of the surface of the moisture-sensitive material to detect humidity [16]. Moisture-sensitive materials are the core of humidity sensors and mainly include polymers [17], ceramics [18], metal oxide semiconductors [19], carbon nanomaterials and their composite [20,21]. In today’s society, where science and technology are developing rapidly, there is an increasing need to measure humidity in different environments, for example, cement, metal smelting, food processing, etc., where humidity at different temperatures needs to be measured and controlled. However, the conductivity and water absorption of conventional materials change very easily at different temperatures, and such changes are often irreversible. Therefore, the application of humidity sensors at different temperatures is greatly limited. However, with the development of nanomaterials, excellent nanomaterials such as graphene and carbon nanotubes are also used as humidity-sensitive materials. In recent years, the two-dimensional nanomaterial graphene has attracted increasing attention in the field of humidity sensing because of its high specific surface area, excellent mechanical strength, large signal-to-noise ratio, extremely high absorption coefficient, and high charge carrier mobility [22,23,24,25,26].

Since 2004, Novoselov, k.s. and Jim. first isolated graphene from graphite using micromechanical methods at the University of Manchester, UK. Graphene is considered one of the most promising materials due to its excellent electrical properties. In recent years, many researchers have modified graphene by reductive oxidation, which can change the properties of graphene so that it can be used to measure humidity [27], gases [28], temperature [29], etc. In 2017, D. Toloman [30] modified graphene with tin oxide to fabricate highly responsive humidity sensors. In 2018, Shojaee et al. [31] prepared partially reduced graphene oxide (PRGO) nanosheets on polyimide substrates by hydrothermal method to prepare sensor structures with gold interdigital electrodes. The humidity sensitivity performance was also investigated, and the sensor was found to have a fast response/recovery time and high flexibility to monitor human respiration in real-time. In 2019, Xing et al. [32] fabricated a graphene mesh structure covered on the surface of micro-nano fibers to make a humidity sensor with high humidity sensitivity. Based on this, we used rGO-SDS composite film as the humidity sensing material and prepared interdigital electrodes (IDE) by MEMS process. Finally, the moisture-sensitive material was drop-coated on the interdigital electrode, and the sensor characteristics were tested at different temperatures and different humidity levels rGO-SDS.

## 2. Experimental and Computational Section

### 2.1. Design of Sensor

#### 2.1.1. Sensor Size Design

**Figure 1:**
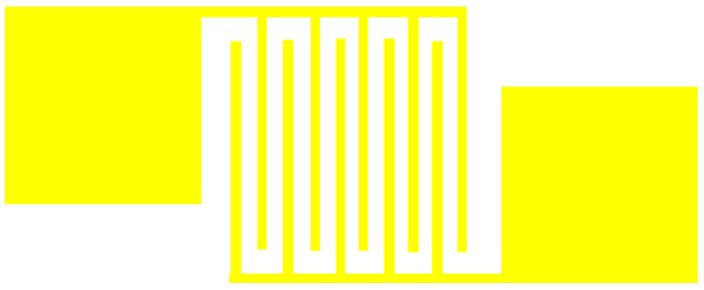
The style of IDE.

Interdigital electrode (IDE) is one of the most common electrode structures, which has advantages of small size, low energy consumption and low cost. It has been widely used in thin film-type gas sensors and humidity sensors. However, the size of IDE is very important. After reviewing the data, we finalized the style and size of the cross-fingers electrode, the style of IDE is shown in Figure 1, and the electrode parameters of IDE are shown in Table 1.

#### 2.1.2. The Preparation of the Sensor

**Figure 2:**
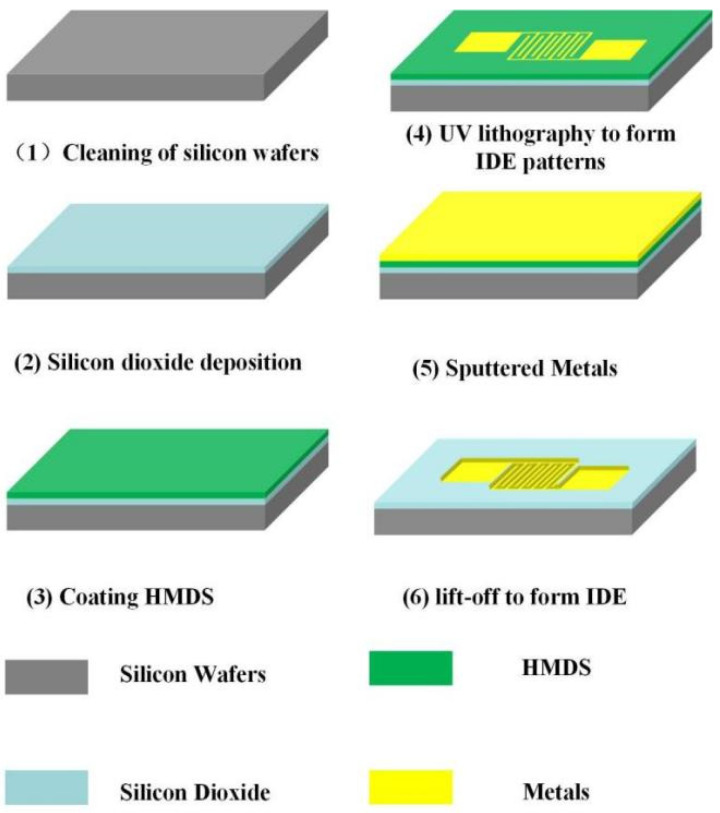
Preparation process of IDE.

The preparation of the sensor mainly includes the preparation of IDE and moisture-sensitive material. For the IDE, we choose standard MEMS technology. In terms of materials, a single cast silicon wafer with a thickness of 525 μm was used as the substrate. The following steps are the key processes for the preparation of the IDE. First, the silicon wafer is cleaned for 15 min in a strong acid solution, which consists of hydrogen peroxide mixed with concentrated sulfuric acid in a 1:3 volume ratio of hydrogen peroxide to concentrated sulfuric acid, and the strong surface acid solution of the silicon is rinsed with deionized (DI) water. Then it was cleaned for 5 min in a strong alkali solution, which consisted of hydrogen peroxide mixed with ammonium hydroxide and DI water in a 2:1:7 volume ratio of hydrogen peroxide to ammonium hydroxide to DI water, and the strong surface alkali solution of the silicon was rinsed with DI water. The above cleaning steps complete the cleaning of the silicon surface. Second, a layer of silicon dioxide with a thickness of 200 nm is deposited on the cleaned silicon wafer by plasma-enhanced chemical vapor deposition (PECVD) technology. This layer acts as an insulating layer between the metal electrode and the substrate. Third, IDE was formed on the surface of SiO_2_ by ultraviolet lithography. In the lithography progress, in order to increase the stickiness between optical resist and SiO_2_ surface, we coated the Hexamethyldisiloxane (HMDS) adhesion agent on the surface of silicon in a high temperature before lithography. Later, using magnetron sputtering technology, we deposited a metal layer with a thickness of 200 nm on the prepared substrate. In order to remove the excess metal, the deposited metal substrates were subjected to ultrasonic stripping in acetone and alcohol solution for 5 min, respectively. The above is the completion of IDE preparation, as shown in Figure 2.

**Figure 3:**
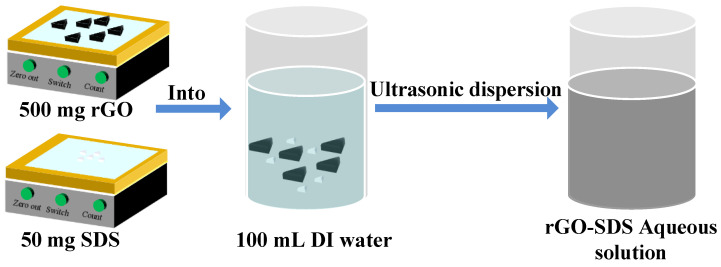
Preparation of moisture-sensitive materials.

In the experiment, we used rGO-SDS composite as the moisture-sensitive material. First, we weighed 0.5 g of RGO with purity greater than 99 wt% and 0.05 g of sodium dodecyl sulfonate (SDS) with an electronic balance. SDS as a dispersant can effectively reduce the deposition of rGO. Then, the weighed rGO and SDS were poured into a beaker filled with 100 mL DI water and dispersed in an ultrasonic cleaning machine for 1 h. Finally, a well-mixed rGO-SDS aqueous solution with a concentration of 5 mg/mL was obtained. The process for moisture-sensitive material is shown in Figure 3.

**Figure 4:**
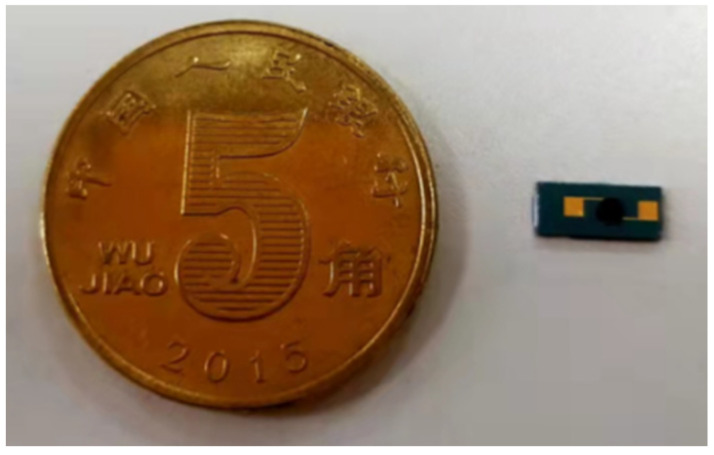
The physical diagram of the sensor.

Finally, an appropriate amount of rGO-SDS aqueous solution was drawn with a micro-sampling needle, drop-coated on the electrode so that the aqueous solution covered the electrode, and the IDE was placed on a 100 °C constant temperature hot plate to dry to prepare a humidity-sensitive film. The above completes the preparation of the sensor. The physical diagram of the sensor is shown in Figure 4.

#### 2.1.3. Characterization of Moisture Sensitive Material

##### Scanning Electron Microscope Characterization

**Figure 5:**
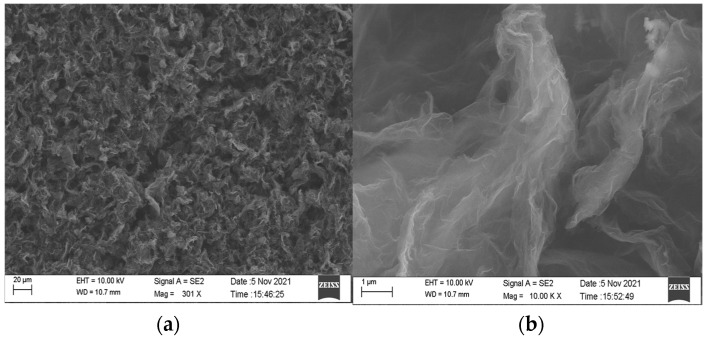
(**a**) SEM image of rGO-SDS composite film. (**b**) Partial enlargement of the majority.

During the baking process of the rGO-SDS humidity-sensitive film prepared by the drop-coated method, the high-temperature heating solution causes the water to evaporate, leaving the sensitive material attached to the electrode and the surface of the sensitive film wrinkled. In order to characterize the surface morphology of the moisture-sensitive film, the IDE coated with rGO-SDS composite film was observed by scanning electron microscopy (SEM), and the results are shown in Figure 5. From the figure, we can observe that the sample surface on the IDE is uniformly dispersed and folded, which greatly increases the contact area between water molecules and graphene, thus further improving the performance of the sensor.

##### Raman Spectroscopy Characterization

**Figure 6:**
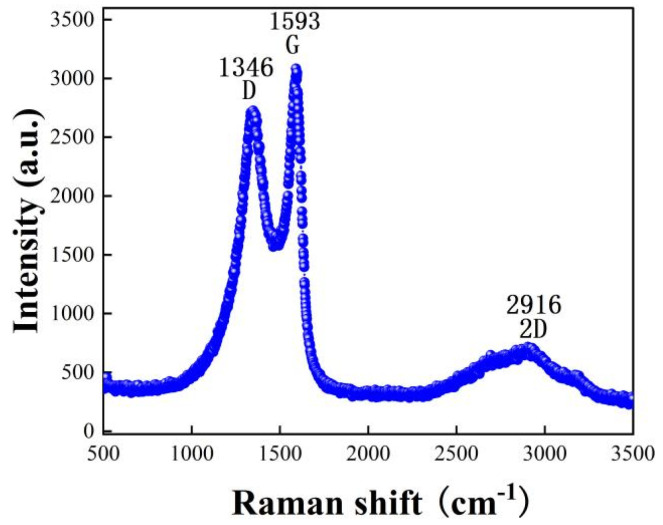
Raman spectra of rGO-SDS composite film.

Raman spectroscopy is another effective tool to study carbon nanomaterials [33]. We observed the characteristic peaks of the moisture-sensitive materials by Raman spectroscopy, and the results are shown in Figure 6. From the figure, we can observe that 1346 cm^−1^ is the D peak: indicates the peak of sp^3^ hybridized defective state C, and 1593 is the G peak indicates the peak of sp^2^ hybridized graphite C. The ratio of D-band to G-band intensity (I_D_/I_G_) is used to quantify the defects present in a particular graphene sample [34,35]. I_D_/I_G_ = 7.4/8.6 = 0. 86, I_G_ is greater than I_D_, indicating that graphene has few lattice defects and low disorder, while the predominance of sp^2^ hybrid graphite C=C. The G peak appearing near 1593 cm^−1^ originates from the plane vibration of the first-order E2g phonon, which reflects the symmetry and order of the material; the 2D peak near 2916 cm^−1^ is a two-phonon resonance Raman peak, whose intensity reflects the stacking degree of graphene. The more layers of graphene, the stronger the sp^2^ vibration of carbon atoms and the higher the G-peak. Graphite layers below five layers can be determined by Raman spectroscopy, and in particular, the two-dimensional peaks can be used to distinguish single-layer graphene sheets from multilayer graphene sheets. The 2D peaks are about 30 cm^−1^ wide for single-layer graphene flakes, 50 cm^−1^ wide for bilayer graphene flakes, and wider for more than three layers, but the difference is not significant. I_2D_/I_G_ = 2.1/8.6 = 0.244, indicating that the intensity of the 2D peaks is weak, and in general graphene, the intensity of the 2D peaks is greater than that of G.

##### Atomic Force Microscopy Characterization

**Figure 7:**
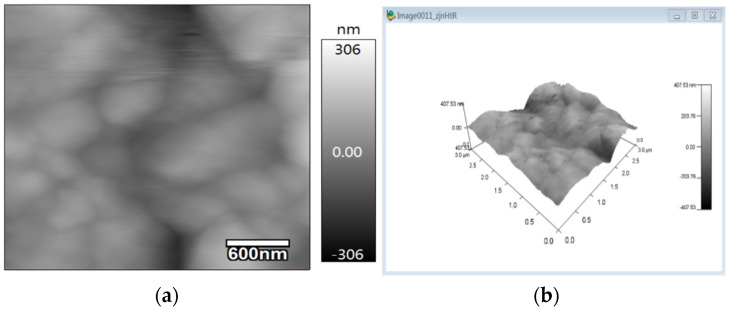
Atomic force microscopy micrographs. (**a**) Two-dimensional surface morphology of rGO-SDS composite film. (**b**) 3D surface roughness of rGO-SDS composite film.

As shown in Figure 5, there are wrinkles on the surface film of IDE samples drop-coated with rGO-SDS, and the generated wrinkles can increase the surface area of the moisture-sensitive material. By characterizing the roughness of the moisture-sensitive material with atomic force microscopy (AFM), the IDE coated with the rGO-SDS composite was observed, as shown in Figure 7, and it was found that the surface roughness was low, and the arithmetic mean profile deviation Ra was 44.913 nm, relative to the profile The root mean square value Rq of the mean line deviation was 57.689 nm.

##### Infrared Spectroscopy 

**Figure 8:**
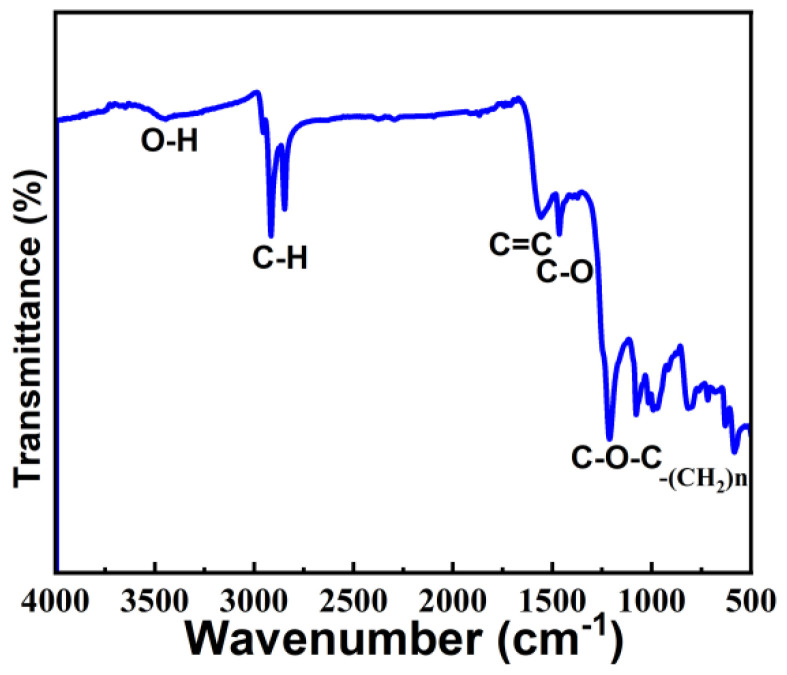
Infrared spectroscopy of rGO-SDS composite film.

To study the structure and chemical bonding of moisture-sensitive material, we performed infrared spectroscopy (FTIR), as shown in Figure 8, The moisture-sensitive material was found to contain OH, CH, C=C, C-O, C-O-C, and -(CH_2_)n bonds. The value 3400 cm^−1^ corresponds to the characteristic absorption peak of OH, but the peak is very weak, indicating low OH content. The values 2955 and 2869 cm^−1^ correspond to the stretching vibration of CH bonds, 1589 cm^−1^ corresponds to the stretching vibration of C=C, 1485 cm^−1^ corresponds to the bending vibration peak of C-O bonds, and the 950–1250 cm^−1^ band corresponds to the stretching vibration peak of C-O-C bonds. FTIR shows that the rGO in moisture-sensitive materials is partially reduced, and the material is rich in oxygen functional groups.

##### X-ray Photoelectron Spectroscopy 

**Figure 9:**
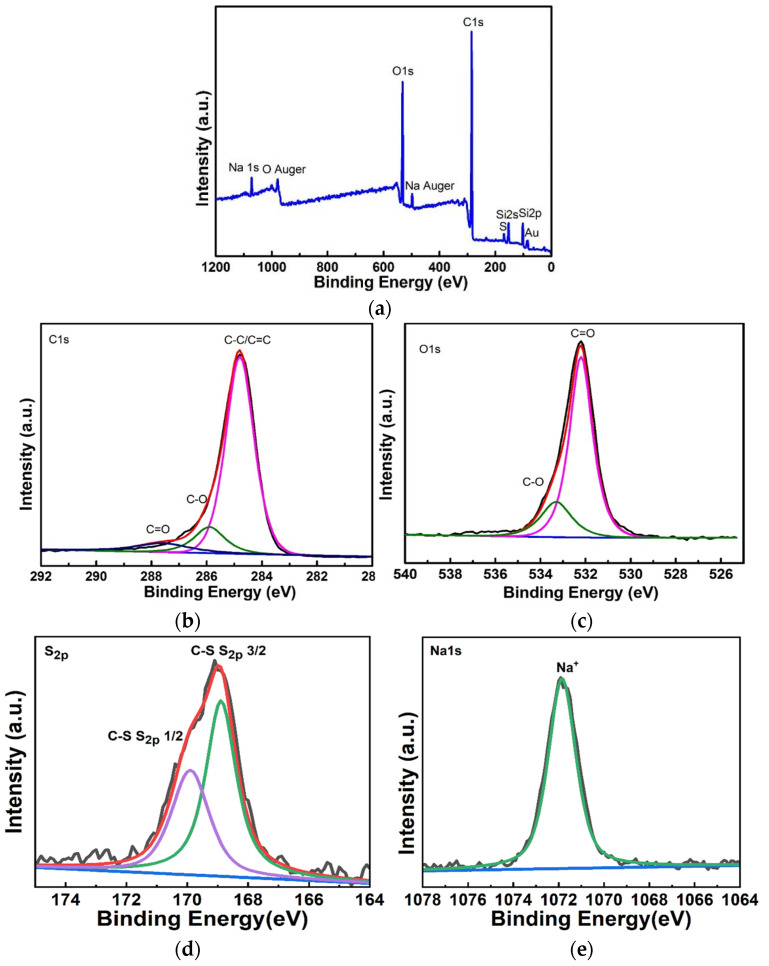
(**a**) The full spectrum of the XPS of the rGO-SDS composite film. (**b**,**c**) XPS fine spectrum of element C and O of rGO-SDS composite film. (**d**,**e**) XPS fine spectrum of element S and Na of rGO-SDS composite film.

Since FTIR cannot quantitatively characterize each functional group, the content of each functional group cannot be calculated, we performed X-ray photoelectron spectroscopy (XPS) analysis on the moisture-sensitive materials. As shown in Figure 9a, the full spectrum of xps. It was found that the moisture-sensitive material has elements of C, O, S, Na. And C and O are the elements of rGO, S and Na are the elements of SDS. In addition, Au is the element of the metal layer, Si is the element of silicon wafer, and SiO_2_ is the element of the insulating layer.

For a detailed analysis of C, O, S and Na elements, we created the xps fine spectra of the corresponding elements, as shown in Figure 9b–e. From the analysis, we obtained that C in the moisture-sensitive material mainly exists as C-C/C=C bond, and the content ratio of C-C/C=C, C-O and C=O is 80.2%:12.3%:7.5%; the presence of C elements mostly as C-C/C=C bonds and less as C-O, C=O indicates that there are few defects in rGO in our moisture-sensitive materials, which is consistent with those detected by Raman spectroscopy. O mainly exists as C=O, and the content ratio of C=O to C-O is 79.3%:20.7%. S mainly exists as C-S and S_2P_. Na is mainly present as Na^+^.

## 3. Results and Discussion

### 3.1. Test Systems

**Figure 10:**
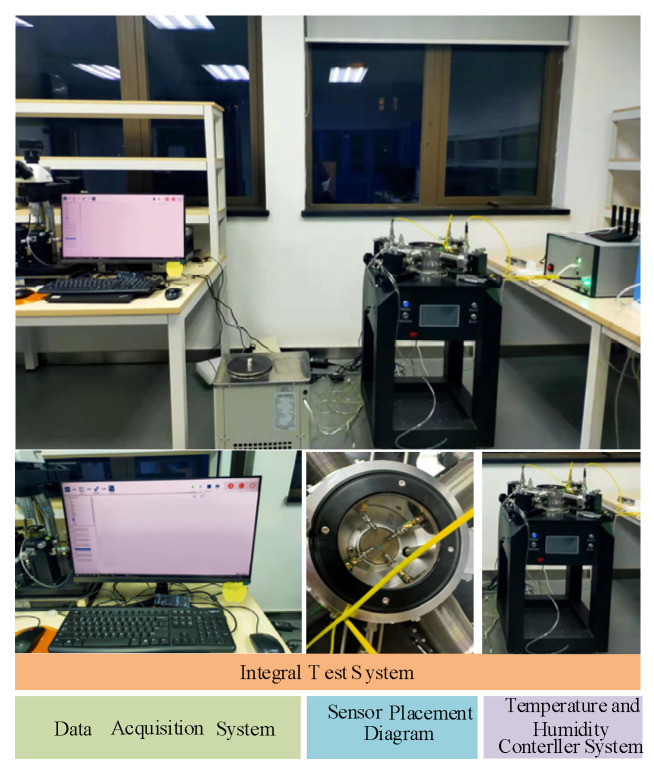
Diagram of the sensor test device.

In order to realize the real-time dynamic testing of the sensor under different humidity and temperatures, a test system was constructed as shown in Figure 10, which consists of three main parts: humidity sensitive controller, temperature controller and data acquisition system. In the experiment, the sensor is placed horizontally in a closed device, and the response of the sensor under different parameters is tested by changing the humidity and temperature of the environment where the sensor is located.

### 3.2. The Sensitivity Test

The principle of humidity sensors is mainly that the adsorption of H_2_O water molecules by moisture-sensitive materials causes the movement of surface electrons, resulting in changes in their conductivity. rGO contains a large number of oxygen-containing groups, and SDS is a good dispersant with good stability. SDS as a dispersant can improve the water solubility of rGO materials, making rGO disperse uniformly in water [36]. The humidity-sensitive membrane prepared by SDS treatment was evenly distributed on the electrode. rGO is a typical p-type semiconductor whose conductivity is controlled by holes [37,38]. At low relative humidity, water molecules bind to the adsorption sites of oxygen-containing groups on the rGO-SDS composite film surface through hydrogen bonding, when the water molecules are free electron donors and cannot move freely, leading to a decrease in the whole density of reduced graphene oxide, which is manifested as an increase in film resistance [39,40]. At high relative humidity, with the increase in humidity, water molecules become mobile and can ionize to produce H_3_O^+^ as a charge carrier. The hydration of H_3_O^+^ is very favorable in liquid water, and the hydration reaction of H_3_O^+^ occurs in the case of enough adsorbed H_2_O, H_2_O + H_3_O^+^ → H_3_O^+^+ H_2_O, and according to the ion transfer mechanism, energy conservation, so the transfer of H_3_O^+^ occurs very easily. As shown by Raman spectroscopy, our prepared rGO-SDS composite film is a multilayer graphite sheet, which is prone to interlayer expansion effects, and the expansion behavior in turn increases the interlayer distance between the rGO-SDS composite film, leading to an increase in film resistance [41]. In addition, rGO-SDS composite film provides better carrier mobility [42]. At high temperatures, there is a thermal generation of excess carriers between the hole-carrying valence band and the electron-carrying conduction band in rGO-SDS composite film, which alters the charge transport in rGO-SDS; for rGO-SDS composite film, the transition from the impurity/defect state to the hole-carrying valence band and the electron-carrying conduction band depends on the location and temperature of the defect level, and the rGO-SDS composite film depends on the charge-carrying scattering of electron phonons and phonons in the rGO-SDS composite film. Therefore, the resistance of the rGO-SDS composite film varies greatly with temperature [43,44].

**Figure 11:**
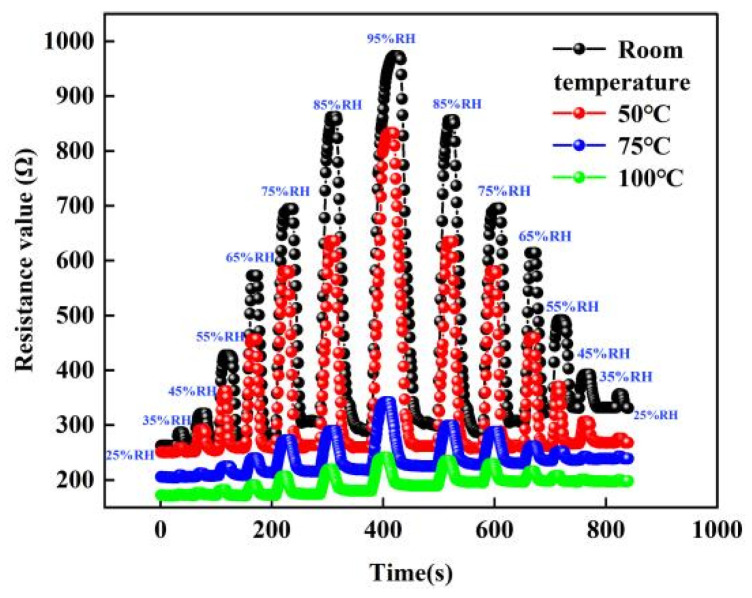
The resistance change curve of sensor at different temperature and humidity environments.

For the experiments of sensitivity testing, we chose to place the sensor in a closed environment and changed the humidity of the sensor by a humidity controller. In addition, we control the temperature of the sensor with a heating system and select the temperature of the sensor as room temperature, 50, 75 and 100 °C, respectively. When the sensor is selected at a certain temperature environment, the humidity environment of the sensor is changed by the humidity-sensitive controller. The humidity in the environment is 25% RH. When the humidity of the sensor increases from 25% RH to 95% RH and then decreases to 25% RH, the change in the resistance value of the sensor is tested, as shown in Figure 11 below. From the graph, it can be seen that the resistance value of the sensor increases with the increase in humidity. This is because as the humidity increases, the number of water molecules adsorbed by the graphene film gradually increases. Finally, while the humidity decreases, the resistance changes to the opposite trend. In addition, we can find that the change in the resistance of the sensor decreases with the increase in temperature.

**Figure 12:**
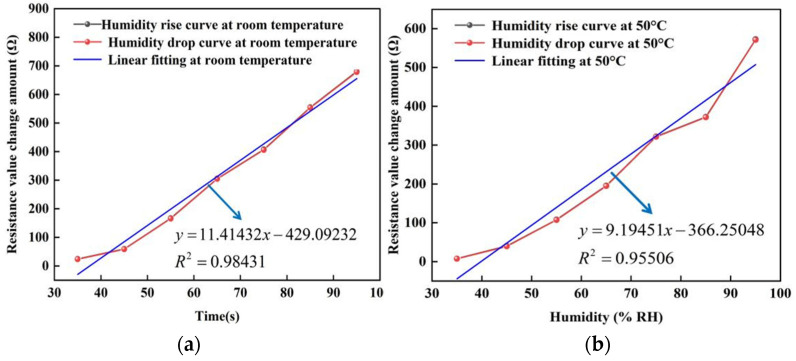

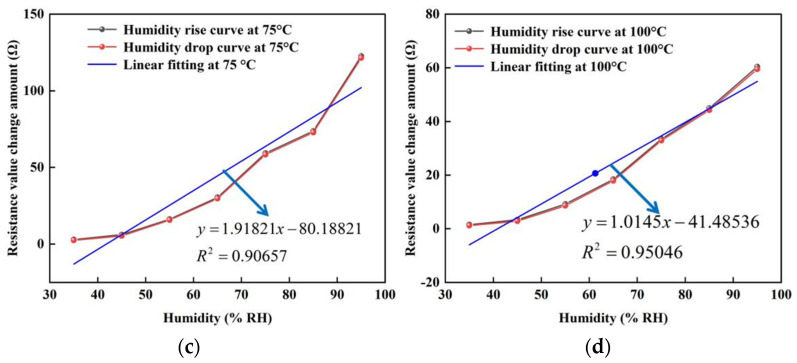
The resistance change curve of the humidity sensor at different temperatures. (**a**) At room temperature. (**b**) At 50 °C. (**c**) At 75 °C. (**d**) At 100 °C.

In addition, the stable resistance of the sensor under different humidity environments was also selected to fit the pictures of humidity and resistance changes at room temperature, 50, 75 and 100 °C, respectively. In Figure 12, the black line represents the curve of rising humidity, the red line represents the curve of falling humidity, and the green line represents the correlation between humidity and sensor resistance after fitting. As can be seen in the figure, the black and red curves refer to the high overlap during the rise and fall of humidity, which indicates that the change in sensor resistance is constant. This is because the water molecules adsorbed on the surface of the graphene film are invariant in a humid environment. In addition, it can be found from the fitted curves that the change in humidity is linearly related to the change in resistance over the ambient humidity range of 25–95% RH. At 100 °C, the correlation of the sensor is about 0.95046 with a sensitivity of about 1.0145 Ω/% RH; while at room temperature, the sensor has a sensitivity of about 11.41432 Ω/% RH and a linearity of about 0.98431. The hysteresis values between the adsorption and desorption humidity maps shown in Figure 12 are (a): 0.04852%, (b): 0.07219%, (c): 0.11419%, (d): 0.10093%.

### 3.3. The Response-Recovery Time Test

The response time is the time it takes for the sensor signal to rise from zero to a certain percentage of the ventilation equilibrium point. It is usually described by T90. The time required to rise from zero to 50% of the equilibrium signal value is called T50, and the time required to rise from zero to 90% is called T90.

Recovery time is a parameter that expresses how quickly the signal returns when the sensor returns from standard gas to zero gas. Commonly used is RT90, which means the time it takes to recover from the sensor ventilated equilibrium state to a 10% signal.

#### 3.3.1. The Response-Recovery Test at Different Humidity

**Figure 13:**
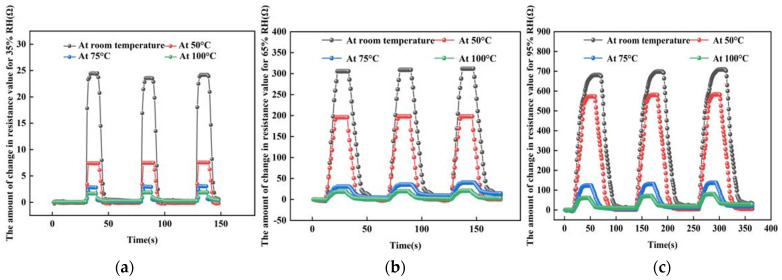
The response-recovery line tests at different humidity environments. (**a**) At 35% RH. (**b**) At 65% RH. (**c**) At 95% RH.

In the experiment, three different humidity environments were chosen to test the response recovery time of the sensor. Figure 13 shows the test curves at 35% RH, 65% RH and 95% RH humidity environments, respectively. In each humid environment, the response recovery curves were tested at four temperatures, respectively.

#### 3.3.2. The Response-Recovery Time Test at Different Humidities

**Figure 14:**
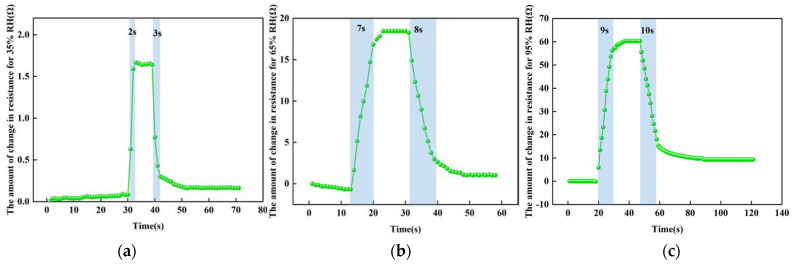
The test results at different humidity levels. (**a**) At 35% RH. (**b**) At 65% RH. (**c**) At 95% RH.

For the sensor response-recovery time tests at different humidity levels, we used a controlled univariate approach, choosing a temperature of 100 °C and humidity levels of 35% RH, 65% RH and 95% RH. Figure 14 shows the test results at different humidity levels. At 100 °C, the response time of the sensor from 25% RH to 35% RH is about 2 s and the recovery time is about 3 s; the response time of the sensor from 25% RH to 65% RH is about 7 s and the recovery time is about 8 s; the response time of the sensor from 25% RH to 95% RH is about 9 s and the recovery time is about 10 s. The higher the humidity, the more water molecules adsorbed by the reduced graphene oxide, the slower the charge transfer, and the slower and longer the rate of absorbing and desorbing water molecules at the same temperature.

#### 3.3.3. The Response-Recovery Time Test at Different Temperature

**Figure 15:**
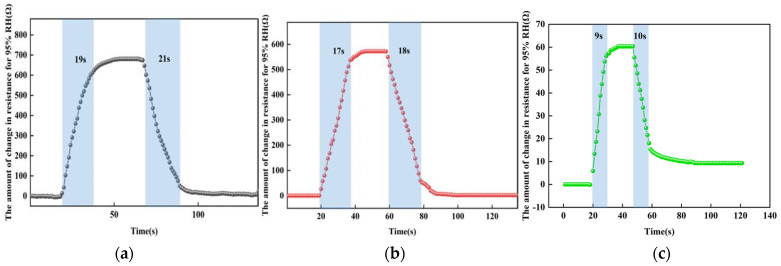
The response-recovery time tests at different temperatures. (**a**) At room temperature. (**b**) At 50 °C. (**c**) At 100 °C.

For the reaction-recovery time tests at different temperatures, we used a controlled univariate approach with temperatures taken as room temperature, 50 °C, and 100 °C. A humidity of 95% RH was selected for the sensor reaction phase and 25% RH for the recovery phase. Figure 15 shows the test results at different temperatures. At room temperature, the response time of the sensor from 25% RH to 95% RH is about 19 s and the recovery time is about 21 s; at 50 °C, the response time of the sensor from 25% RH to 95% RH is about 17 s and the recovery time is about 18 s; at 100 °C, the response time of the sensor from 25% RH to 95% RH is about 9 s and the recovery time is about 10 s. The higher the temperature, the higher the reduced graphene oxide, the faster the charge transfer, the faster the rate of absorbing and desorption of water molecules under the same humidity, and the shorter the time.

### 3.4. The Repeatability Test

**Figure 16:**
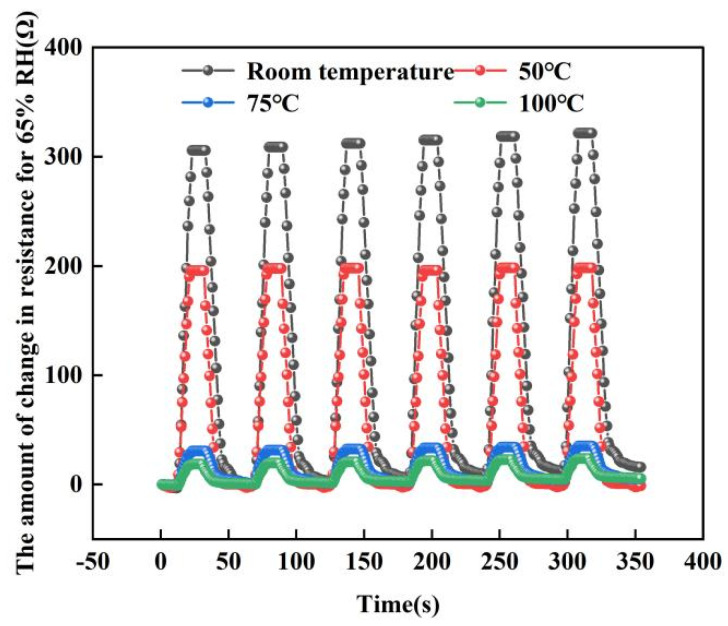
The repeatability test at different temperatures (At 65% RH).

For repeated measurement experiments, we chose to place the sensor into the test system and run the humidity from 25% RH to 65% RH for six cycles. Four tests were performed at room temperature, 50, 75 and 100 °C. Figure 16 below shows a graph of the test results. From the graph, the sensor maintained good repeatability in six repeatability tests. In addition, we can find that: as the temperature increases, the resistance of the sensor decreases. At room temperature, the resistance of the sensor changes up to 320 Ω, but when the temperature rises to 100 °C, its resistance changes only 19 Ω. This can further explain that the response of the sensor will be weakened to a certain extent in high-temperature environments. This is because at low temperatures, the change in sensor resistance is caused by the change in ambient humidity. In this state, the transfer of electrons on the graphene surface is mainly achieved by the adsorption of water molecules. However, when the temperature increases, a large amount of electron transfer occurs on the graphene surface, so the amount of electron transfer decreases when the humidity changes at high temperatures. 

### 3.5. The Stability Test

**Figure 17:**
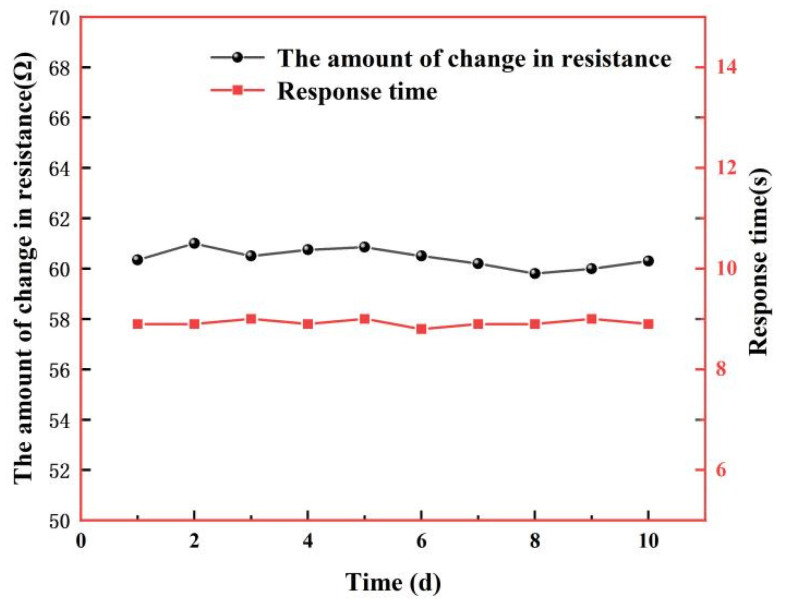
Stability test at 100 °C, 95% RH over 10 days.

To test the stability of the sensor, we placed the sensor in the above test setup with the temperature set at 100 °C and humidity at 95% RH, measured the sensor for 10 days, and analyzed the changes in response values and response times over the 10-day period. Figure 17 shows the test results. From the figure, it can be found that the changes in resistance value and response time during the ten-day test at 100 °C and 95% RH conditions are almost unchanged within the error range, indicating that the sensor has good stability.

### 3.6. Comparted with the Recently Published Papers on Humidity Sensors

In order to objectively represent the performance of the rGO-SDS humidity sensor, a comparison Table 2 has been added to compare the device in the manuscript with recently published papers on humidity sensors in order to compare its performance with them.

## 4. Summary

In this paper, a humidity sensor was designed and prepared with rGO-SDS composite film as the moisture-sensitive material, and its key structure of interdigital electrode was prepared by a microelectromechanical system (MEMS) process, and the sensor was tested at different temperatures and different humidity levels. The results show that the humidity variation of the sensor is linearly related to the change of resistance in the ambient humidity range of 25–95% RH. At room temperature, the linearity of the sensor is about 0.98431, and the sensitivity is about 11.41432 Ω/% RH; at 100 °C, the correlation of the sensor is about 0.95046, and the sensitivity is about 1.0145 Ω/% RH with short response time and recovery time; its repeatability is good in six consecutive cycles of experiments from 25% RH to 65% RH at room temperature, 50, 75 and 100 °C; at 100 °C, 95% RH test for 10 days, the resistance change and response time remained essentially constant within the error range. Currently, the sensor is directly heated, and subsequently, heated electrodes can be designed between the silicon substrate and the insulating layer to facilitate the operation of the sensor in the constant temperature range.

## Figures and Tables

**Table 1:** The electrode parameters of IDE.

The Amount of IDE	The Length of IDE	The Width of IDE	The Distance between Interdigital
5	520 μm	30 μm	30 μm

**Table 2:** Compared with the recently published papers on humidity sensors.

Active Material	Response Time (s)	Recovery Time (s)	Range	Average Sensitivity (X/% RH)	Reference
rGO-SDS	9	10	25–95%	1.0145 Ω/% RH	This Work
GrF/ZnO	0.4	4	15–86%	7.77 µA/% RH	[45]
sodium hyaluronate (SH)/multi-walled car-bon nanotubes	0.32	0.27	11–98%	/	[46]
P(VDF-T rFE)/Graphene-Flower	0.8	2.5	8–98%	0.0558 pF/% RH	[47]
Biocompatible Egg White Thin Film	1.2	1.7	10–85%	50 kΩ/% RH	[48]
MoS_2_/Graphene Oxide/C60-OH Nanostructures	/	/	11–97%	/	[49]
